# Assessment of Mitochondrial Dysfunction and Monoamine Oxidase Contribution to Oxidative Stress in Human Diabetic Hearts

**DOI:** 10.1155/2016/8470394

**Published:** 2016-04-13

**Authors:** O. M. Duicu, R. Lighezan, A. Sturza, R. Balica, A. Vaduva, H. Feier, M. Gaspar, A. Ionac, L. Noveanu, C. Borza, D. M. Muntean, C. Mornos

**Affiliations:** ^1^Department of Pathophysiology, “Victor Babeș” University of Medicine and Pharmacy, 2 Eftimie Murgu Square, 300041 Timișoara, Romania; ^2^Center for Translational Research and Systems Medicine, “Victor Babeș” University of Medicine and Pharmacy, 2 Eftimie Murgu Square, 300041 Timișoara, Romania; ^3^Department of Parasitology, “Victor Babeș” University of Medicine and Pharmacy, 2 Eftimie Murgu Square, 300041 Timișoara, Romania; ^4^Department of Histology, “Victor Babeș” University of Medicine and Pharmacy, 2 Eftimie Murgu Square, 300041 Timișoara, Romania; ^5^Department of Morphopathology, “Victor Babeș” University of Medicine and Pharmacy, 2 Eftimie Murgu Square, 300041 Timișoara, Romania; ^6^Department of Cardiovascular Surgery, “Victor Babeș” University of Medicine and Pharmacy, 2 Eftimie Murgu Square, 300041 Timișoara, Romania; ^7^Department of Cardiology, 2nd Cardiology Clinic, “Victor Babeș” University of Medicine and Pharmacy, 2 Eftimie Murgu Square, 300041 Timișoara, Romania

## Abstract

Mitochondria-related oxidative stress is a pathomechanism causally linked to coronary heart disease (CHD) and diabetes mellitus (DM). Recently, mitochondrial monoamine oxidases (MAOs) have emerged as novel sources of oxidative stress in the cardiovascular system and experimental diabetes. The present study was purported to assess the mitochondrial impairment and the contribution of MAOs-related oxidative stress to the cardiovascular dysfunction in coronary patients with/without DM. Right atrial appendages were obtained from 75 patients randomized into 3 groups: (1) Control (CTRL), valvular patients without CHD; (2) CHD, patients with confirmed CHD; and (3) CHD-DM, patients with CHD and DM. Mitochondrial respiration was measured by high-resolution respirometry and MAOs expression was evaluated by RT-PCR and immunohistochemistry. Hydrogen peroxide (H_2_O_2_) emission was assessed by confocal microscopy and spectrophotometrically. The impairment of mitochondrial respiration was substrate-independent in CHD-DM group. MAOs expression was comparable among the groups, with the predominance of MAO-B isoform but no significant differences regarding oxidative stress were detected by either method. Incubation of atrial samples with MAOs inhibitors significantly reduced the H_2_O_2_ in all groups. In conclusion, abnormal mitochondrial respiration occurs in CHD and is more severe in DM and MAOs contribute to oxidative stress in human diseased hearts with/without DM.

## 1. Introduction

Coronary heart disease (CHD) represents the most important cause of mortality and morbidity attributable to heart failure worldwide. Progression of the disease is aggravated by diabetes mellitus (DM), a major independent risk factor, whose prevalence is alarmingly high [1]. It is widely accepted that oxidative stress is a major contributor to the pathogenesis of both cardiovascular and metabolic disorders and mitochondria are the principal sources of reactive oxygen species (ROS) (recently reviewed in [2]). In this respect, the role of the electron transport chain (ETC) at the inner mitochondrial membrane as the critical site for ROS production in the failing rat myocardium has been already reported in the late 90s. Indeed, these authors reported a decrease in complex I activity as the major reason responsible for the deleterious ROS production [3] that has been further correlated with left ventricular contractile dysfunction [4]. Since then, a substantial body of research has been carried out in order to shed light on the impairment of mitochondrial function in experimental models of heart failure and the failing human heart (recently reviewed in [5–8]).

Similarly, in type 2 diabetes mellitus (T2DM), mitochondrial abnormalities have been reported to accelerate the progression of insulin resistance* via* ROS overproduction (reviewed in [9–11]). Interestingly, in a recent study, the impairment of mitochondrial function and dynamics has been associated with contractile dysfunction in diabetic (but not obese) patients [12]. However, despite the widely reported role of mitochondria-related oxidative stress in diabetes [13, 14], the sources of ROS generation in the heart remain elusive. In particular, the membrane permeable hydrogen peroxide (H_2_O_2_) has been regarded both as signalling molecule and harmful ROS in several pathologies, including diabetes [15, 16].

In the past decade, monoamine oxidases (MAOs) with 2 isoforms (A and B) at the outer mitochondrial membrane have emerged as sources for constant H_2_O_2_ generation in heart and vessels (for a recent comprehensive review see [17]). These flavoproteins catalize the transfer of electrons from the endogenous and dietary amines to O_2_ according to the general reaction: R-CH_2_-NH_2_ + O_2_ + H_2_O → R-CHO + NH_3_ + H_2_O_2_ [18, 19]. Both isoforms are present in the cardiovascular system, MAO-A being largely considered the predominant enzyme in rodents and humans [17]. MAOs-derived H_2_O_2_ contributes to the oxidative stress associated with ischemia/reperfusion injury [20, 21], maladaptive left ventricular hypertrophy [22–24], and endothelial dysfunction in normal and diabetic vessels in rodents [25, 26].

Despite the unequivocal role of MAOs as mitochondrial contributors to the obligatory ROS production in the cardiovascular system in experimental settings [17], the role of these enzymes in coronary patients with diabetes, the most frequent chronic metabolic disease associated with increased oxidative stress, has not been addressed so far [5]. Thus, the aims of the present study were to investigate the status of mitochondrial function and the contribution of MAOs to oxidative stress in coronary patients with and without diabetes.

## 2. Material and Methods

The study is in accordance with the ethical principles for medical research involving human subjects from the Declaration of Helsinki. Approval for this study was granted by the Committee for Research Ethics of the University of Medicine and Pharmacy Timisoara, Romania. Seventy-five patients undergoing heart surgery were randomized into 3 groups: (1) Control (CTRL), valvular patients without documented CHD; (2) CHD, patients with documented CHD; and (3) CHD-DM, patients with documented CHD and DM. All patients gave informed consent prior to surgery. Demographic and clinical data and preoperative medication were collected from medical records and are presented in Table 1. Echocardiography was performed with an ultrasonographic system (Vivid 9 General Electric, Milwaukee, WI) and analyzed using commercially available software (EchoPAC; GE Vingmed Ultrasound AS). LVEF was calculated from apical two- and four-chamber views using a modified Simpson's rule [27].

Atrial myocardium was sampled from patients subjected to open-heart surgery once cardiopulmonary bypass was established, by resecting the tip of the right atrial appendage (approximately 20 mg). The atrial samples were further placed in ice-cold buffer A containing 10 mM Ca-EGTA (ethylene glycol tetra-acetic acid) buffer, 0.1 *μ*M free calcium, 20 mM imidazole, 20 mM taurine, 50 mM K-MES (2-(*N*-morpholino)-ethanesulfonic acid), 0.5 mM DTT (dithiothreitol), 6.56 mM MgCl_2_, 5.77 mM ATP (adenosine-5′-triphosphate), and 15 mM phosphocreatine (pH 7.1 adjusted with 5 N KOH at 0°C) and immediately transferred to the laboratory for further experimental procedures. When needed, parts of the atrial biopsies were snap-frozen.

### 2.1. Atrial Tissue Permeabilization Procedure

Dissection and permeabilization of myocardial fibers were performed according to a previously described method [28]. Briefly, after atrial appendage was trimmed by vascular and connective tissue, myocardial muscle bundles were isolated and immersed in ice-cold buffer A. Fiber bundles were further separated mechanically under a microscope, transferred into ice-cold buffer A supplemented with 50 *μ*g/mL of saponin stock solution (5 mg/mL) and shaken by gentle agitation on ice for 30 min. Following saponin permeabilization, samples were quickly transferred into the incubation buffer B (0.5 mM EGTA, 3 mM MgCl_2_·6H_2_O, 60 mM K-lactobionate, 20 mM taurine, 10 mM KH_2_P0_4_, 20 mM HEPES (4-(2-hydroxyethyl)-1-piperazineethanesulfonic acid), 110 mM sucrose, 1 g/L BSA (bovine serum albumin), essentially fatty acid free, pH = 7.1, at 30°C + 280 U/mL catalase lyophilized powder, and 2000–5000 units/mg protein), shaken by gentle agitation for 10 min on ice. Permeabilized fibers were kept in buffer B on ice throughout the respirometry measurements (~4 hours).

### 2.2. Assessment of Mitochondrial Respiration

Mitochondrial measurements were performed for 1–3 mg (wet weight) of permeabilized fibers at 37°C using the Oxygraph-2k system (Oroboros Instruments Corp., Innsbruck, Austria) as previously described [28]. The Substrate-Uncoupler-Inhibitor Titration (SUIT) protocol was as follows: (1) GM_STATE 2_ and S(Rot)_STATE 2_: addition of glutamate (10 mM) + Malate (2 mM) and Succinate (10 mM) + Rotenone (0.5 *μ*M): STATE 2 evaluation for complex I and complex II dependent respiration; (2) D_OXPHOS_: addition of adenosine diphosphate (ADP, D: 5 mM), OXPHOS capacity measurement; (3) c: addition of cytochrome c (10 *μ*M) to evaluate the intactness of the outer mitochondrial membrane; (4) Atr_STATE 4_: addition of atractyloside (0.75 mM), an ADP/ATP mitochondrial translocase inhibitor, STATE 4 measurement; (5) FCCP_ETS_: FCCP (carbonyl cyanide p-(trifluoromethoxy) phenylhydrazone) titrations (0.5 *μ*M/step) in order to measure uncoupled respiration: ETS (electron transport system) capacity; (6) Ama*ROX*: addition of antimycin A (2.5 *μ*M), and a complex III inhibitor: residual oxygen consumption, subtracted from total flux, ROX state. Mitochondrial respiration was corrected for oxygen flux due to instrumental background and ROX.

Citrate synthase (CS) activity was quantified by measuring the rate of 5,5′-dithiobis-(nitrobenzoic acid) reactive reduced coenzyme A at 37°C with a Hitachi F-7000 spectrofluorometer, according to a previously described method (monitoring the reaction of sodium oxaloacetate, acetyl-coenzyme A, and 5,5′-dithiobis-(2 nitrobenzoic) acid at 412 nm) [29].

### 2.3. Evaluation of Oxidative Stress

The spatial distribution of hydrogen peroxide (H_2_O_2_) throughout the atrial sample was determined using the 2′,7′-dichlorofluorescein diacetate (DCF, Sigma-Aldrich D6882) probe according to a previously described technique [30]. Briefly, the atrial fragments were embedded in OCT and snap-frozen. The frozen fragments were cut in 8 *μ*m thick cryosections and put on glass slides. After 3 washes with PBS, 5 minutes each, the cryosections were incubated in the dark with DCF for 30 minutes at room temperature. Excess DCF was washed away by 3 additional washes with PBS. The slides were mounted with Vectashield (Vector Laboratories) and immediately analyzed with the confocal microscope (Olympus Fluoview FV1000). Images were obtained using laser excitation at 488 nm, detection at 519 nm, with a 40x UPLSAPO objective (NA = 0.95). Image analysis was performed using the Icy Bioimage Analysis software [31].

Hydrogen peroxide production was also assessed in atrial samples in the presence versus the absence of MAO inhibitors (30 min preincubation with clorgyline and selegiline, 10 *μ*M) by means of the ferrous iron xylenol orange oxidation (FOX) assay (PeroxiDetect Kit, Sigma-Aldrich) as previously described [25]. The principle of the assay is that peroxides oxidize Fe^2+^ to Fe^3+^ ions at acidic pH; the Fe^3+^ ion will further form a coloured adduct with xylenol orange (XO,3,3′-bis[N,N-bis-(carboxymethyl) aminomethyl]-o-cresolsulfonephthalein, sodium salt) that is measured at 560 nm.

### 2.4. MAO Gene Expression

MAO-A and MAO-B genes expression was evaluated by quantitative real time polymerase chain reaction (RT-PCR), as previously described [26]. The sequence information of the NCBI database (5′-> 3′: human MAO-A fw AGG ACT ATC TGC TGC CAA AC; human MAO-A rev AAG CTC CAC CAA CAT CTA CG; human MAO-B fw GAA GAG TGG GAC AAC ATG AC; and human MAO-B rev CTC CAC ACT GCT TCA CAT AC) was used to design the primers against MAO isoforms. The housekeeping gene and its primers were as follows: EEF2 (fw): 5′-GAC ATC ACC AAG GGT GTG CAG-3′ and EEF2 (rv): 5′-GCG GTC AGC ACA CTG GCA TA-3′, respectively. Total RNA was isolated from atrial samples with the “Total RNA Mini SI Isolation Spin-Kit, Applichem” and used for reverse transcription (Superscript III RT, Invitrogen). The PCR conditions consisted of initial denaturation (95°C, 10 minutes), 40 cycles of denaturation (95°C, 30 seconds), annealing (55°C, 60 seconds), and elongation (72°C, 60 seconds).

### 2.5. MAO Protein Expression

The immunohistochemistry assay was performed as previously described [32]. Briefly, atrial samples were fixed in 4% buffered formalin, embedded in paraffin, and sectioned at 3–5 *μ*m. The slides were then dewaxed and rehydrated and either stained with the usual Hematoxylin-Eosin (HE) or pretreated for immunohistochemistry. A heat-induced epitope retrieval was performed with Novocastra Bond Epitope Retrieval Solution 1, a ready-to-use, citrate based pH 6.0 solution (Leica Biosystems, UK) for 20 minutes. Endogenous peroxidase blocking was realised with 3% hydrogen peroxide for 5 minutes. This step was followed by the 30-minute incubation with the primary antibodies (Novus Biologicals, USA): anti-MAO-A (mouse monoclonal, 1D6, dilution 1 : 300) and anti-MAO-B (rabbit polyclonal, dilution 1 : 50). The Bond Polymer Refine Detection System was applied for visualisation, and 3,3-diamino-benzidine dihydrochloride was applied as chromogen for 10 minutes. The nuclei were counterstained with hematoxylin for 5 minutes. The immunohistochemistry assay was performed with Leica Bond-Max autostainer (Leica Biosystems, Newcastle uponTyne, UK). After staining, the slides were dehydrated and mounted with Canada balsam. Image acquisition and analysis were performed using a Nikon Eclipse E-600 microscope.

#### 2.5.1. Semi-Quantitative Immunohistochemical Analysis

The immunoreactivity was quantified using the hot-spot method from 3 consecutive areas at the magnification of ×200 with a corresponding area of 0.267 mm^2^, at the microscopic scale of 50 *μ*m. The protein expression was scored using a semiquantitative scale for intensity: absent = 0, week = 1, moderate = 2, and strong = 3. The score for each probe was expressed as a mean score after the quantification of protein expression in the muscular layer, endothelial cells of intramyocardial vessels, and the mesothelium of the atrial sample.

### 2.6. Chemicals

All reagents were purchased from Sigma-Aldrich.

### 2.7. Data Analysis

Data analysis was performed by GraphPad Prism version 6.0 for Windows (GraphPad Software, USA). Data were expressed as means ± SEM. For multiple comparisons, one-way ANOVA followed by Bonferroni post hoc analysis was used, differences between groups being considered significant at *p* < 0.05.

## 3. Results and Discussion

Atrial samples were collected from 75 patients with comparable clinical and demographic data, except for hypertension which was more frequent in CHD patients (Table 1). Fasting plasma glucose (FPG) and body mass index (BMI) were significantly higher in diabetic patients as compared to both CTRL and CHD patients, respectively (Table 1). One of the exclusion criteria in our study was heart failure with reduced ejection fraction where mitochondrial dysfunction has been reported [33]. However, despite the fact that we recruited patients with preserved ejection fraction, a lower mean value of LVEF in CHD patients with/without diabetes as compared to the valvular ones was present (Table 1).

Due to different biopsy amount and/or quality, mitochondrial respiration, MAO gene expression, MAO protein expression, and oxidative stress evaluation have been performed on 60, 30, 30, and 45 samples, respectively.

### 3.1. Complex I-Supported Respiration Is Depressed in CHD Patients with and without Diabetes

**Figure 1:**
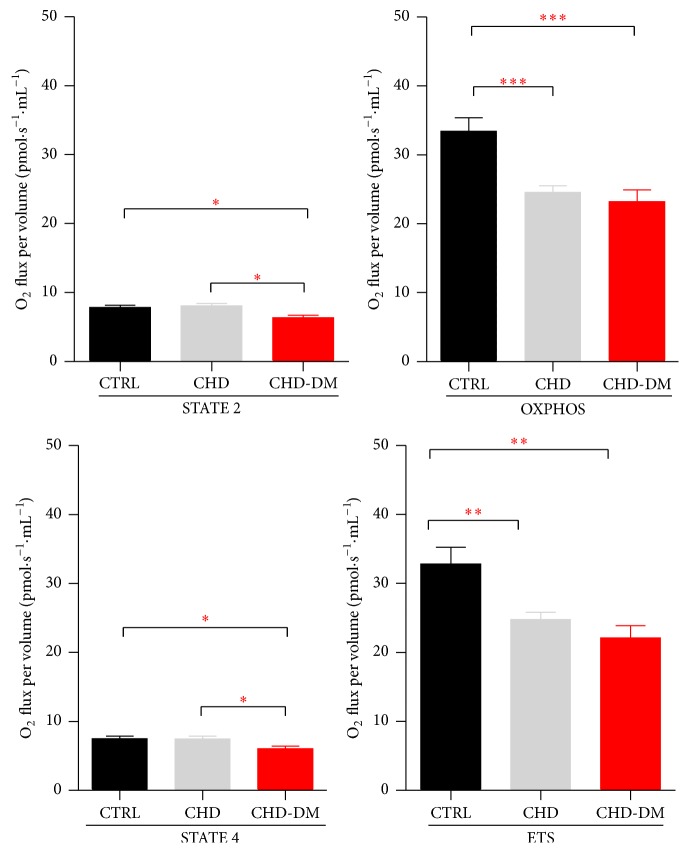
Respiratory parameters for CI-supported respiration (*n* = 20/CTRL group, *n* = 25/CHD group, and *n* = 15/CHD-DM group; values are means ± SEM; ^*∗*^ *p* < 0.05, ^*∗∗*^ *p* < 0.01, and ^*∗∗∗*^ *p* < 0.001).

To characterize mitochondrial dysfunction in coronary patients with and without diabetes, we performed a high-resolution respirometry study in permeabilized atrial fibers using two different SUIT protocols (see Methods). Thus, in mitochondria energized with glutamate + malate we found a significant decrease of OXPHOS and uncoupled respiration (ETS) in coronary patients regardless of the presence of diabetes, whereas STATES 2 and 4 were decreased only in CHD-DM group (Figure 1).

### 3.2. Complex II-Supported Respiration Is Depressed Only in CHD Patients with Diabetes

**Figure 2:**
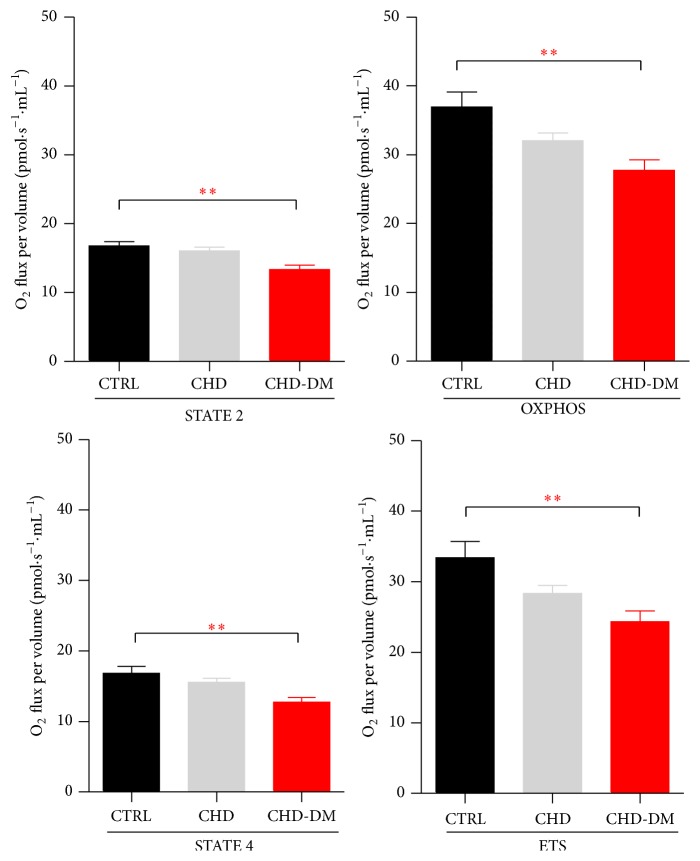
Respiratory parameters for CII-supported respiration (*n* = 20/CTRL group, *n* = 25/CHD group, and *n* = 15/CHD-DM group; values are means ± SEM; ^*∗∗*^ *p* < 0.01).

At variance from the previous findings, in mitochondria energized with succinate (+rotenone) a significant decrease in all respiratory parameters (STATES 2 and 4, OXPHOS, and ETS capacities) was found only in CHD-DM group, suggesting a more significant impairment of mitochondrial function in the presence of diabetes (Figure 2).

We further measured citrate synthase activity (a marker for the content of intact mitochondria) in atrial samples and found comparable values among the three groups (CTRL: 210.2 ± 32.3, CHD: 209.6 ± 21.5, and CHD-DM: 208.8 ± 43.4 nmol·min^−1^·mg^−1^ protein), suggesting that the reduced oxidative phosphorylation in atrial tissue of CHD patients with/without diabetes is not a consequence of the reduction in mitochondrial content.

In the past decades mitochondrial dysfunction and subcellular remodelling associated with heart failure have been systematically characterized in several animal models (reviewed in [34, 35]). Subsequently, an increasing number of studies also described the occurrence of the impairment in mitochondrial oxygen consumption as well as of the increase in ROS production in the hypertrophic/failing human heart [6, 28, 36]. However, it was not clear until recently whether mitochondrial dysfunction is also causally related to the contractile dysfunction in humans. Accordingly, Dela's group reported that left ventricular systolic dysfunction (ejection fraction < 45%) was associated with markedly reduced mitochondrial oxidative phosphorylation when fatty acids were used as substrate [33]. In our previous study performed in patients with preserved ejection fraction (≥50%), we reported the early impairment of complex I-supported respiration without considering the possible contribution of diabetes (since both patients with and without DM were included) [28]. In the present study, we showed that both CI-dependent and CII-dependent respiration is decreased in coronary patients with diabetes and preserved ejection fraction. Our results are in agreement with the study of Anderson et al. who reported a marked decrease in the oxidative capacity for glutamate and fatty acid-supported respiration together with an increased content of myocardial triglycerides in the atrial tissue of diabetic* versus* nondiabetic patients [14]. More recently, in an elegant and comprehensive study performed in 141 patients with/without DM and no sign of cardiomyopathy, Montaigne et al. reported an association between impaired mitochondrial function/dynamics and contractile dysfunction in diabetic (but not in nondiabetic) obese patients and indicated that chronic hyperglycemia is mainly responsible for mitochondrial dysfunction in the diabetic heart [12].

### 3.3. H_2_O_2_ Emission Was Not Increased in Diabetic versus Nondiabetic Patients

**Figure 3:**
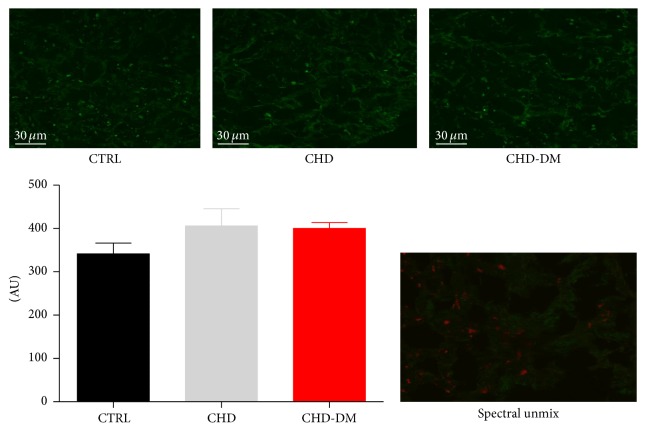
Atrial H_2_O_2_ emission detected with DCF fluorescence production. Fluorescent green positive staining was measured by confocal microscopy in the dichlorofluorescein- (DCF-) treated images. Levels of H_2_O_2_ detected with DCF fluorescence were similar in the studied groups (*n* = 10/group). Spectral unmixing was done using Lambda scan acquisition mode, range 490–600 nm, step 10 nm. The resulting composite image shows the real DCF component (red channel) on the autofluorescent background (green channel).

**Figure 4:**
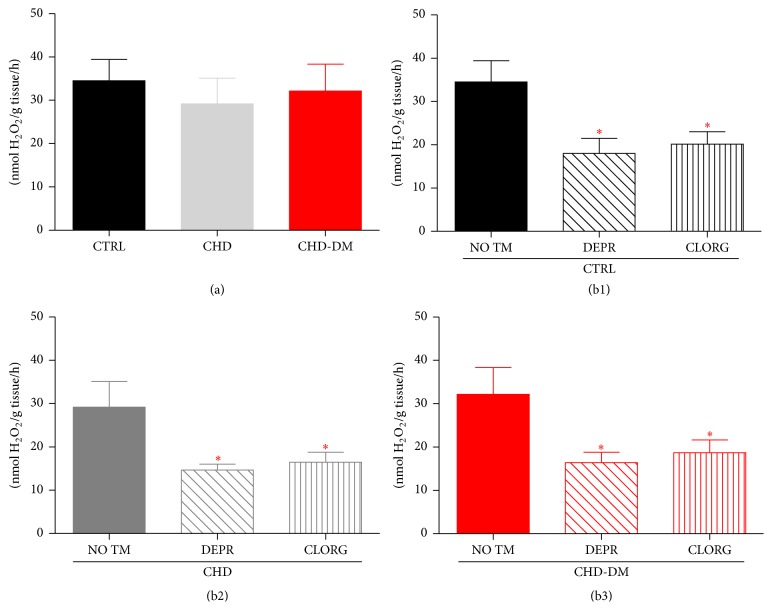
Atrial H_2_O_2_ emission detected with FOX assay. (a) Levels of H_2_O_2_ were similar in all groups (*n* = 5/group). (b1)–(b3) H_2_O_2_ level in the presence of selegiline and clorgyline (10 *μ*M each) versus their corresponding controls (i.e., not treated atrial samples: NO TM) in each of the studied groups (*n* = 5/group; values are means ± SEM; ^*∗*^ *p* < 0.05 versus not treated atrial samples).

To assess the oxidative stress, a classic feature of DM, we measured in atrial samples the H_2_O_2_ release by two methods, confocal microscopy (with the DCF probe) and FOX assay. However, we could not identify a significant increase in hydrogen peroxide in atrial samples isolated from diabetic versus the other 2 groups (Figures 3 and 4(a)). This finding is rather surprising, considering the large body of evidence suggestive for the role of oxidative stress in the evolution of diabetes [37]. Moreover, two recent studies performed in the same model (human atrial samples) reported higher rates of H_2_O_2_ production derived from the mitochondrial electron transport chain (ETC) in diabetic versus nondiabetic patients [13, 14]. These authors estimated the rate of H_2_O_2_ originating from ETC as being at least 10-fold lower than the one derived from either MAOs or the NADPH-oxidases alone [38]. A possible explanation for our unexpected result could be that we included in the CHD-DM group both obese and nonobese patients; whether the diabetic nonobese patients are less prone to develop oxidative stress it is not known. Another possible explanation might be related to the preoperative medication in coronary patients (versus the control group), statins, and angiotensin-converting enzyme inhibitors being known to be able to mitigate the oxidative stress [39].

### 3.4. *Ex Vivo* Inhibition of MAOs Attenuated Atrial H_2_O_2_ Generation in All Groups

In order to assess the possible contribution of MAOs to oxidative stress, we further measured by FOX assay the atrial H_2_O_2_ production in the presence of two irreversible MAO inhibitors: selegiline for MAO-B and clorgyline for MAO-A. As shown in Figures 4(b1), 4(b2), and 4(b3), H_2_O_2_ production was significantly reduced in the presence of both MAOs inhibitors in all groups, an observation that is highly suggestive for the role of MAOs as important sources of oxidative stress in diseased atrial myocardium.

### 3.5. MAO mRNA Expression Was Comparable among the Groups and MAO-B Was the Predominant Isoform

**Figure 5:**
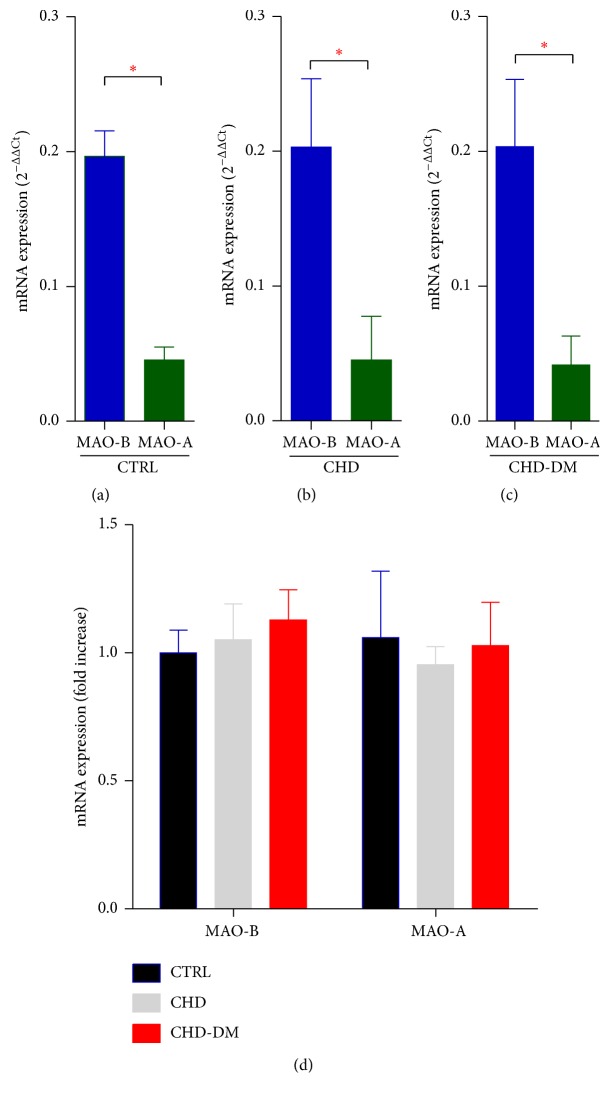
MAO mRNA expression in human atrial samples. (a)–(c) RT-PCR (mRNA expression: 2^−ΔΔCt^) for MAO-A and MAO-B relative to the housekeeping gene EEF2*α* in atrial samples from CTRL group (a), CHD group (b), CHD-DM (c), *n* = 10, ^*∗*^ *p* < 0.05, and (d) RT-PCR (fold increase) for MAO-A and MAO-B relative to the housekeeping gene EEF2*α* in atrial samples (*n* = 10/group).

To evaluate the potential induction of MAOs, we further assessed the expression of MAO-A and MAO-B in human atrial samples by RT-PCR. Interestingly, the expression of MAO-B isoform mRNA (by analyzing the computed threshold value difference of the RT-PCR) was more abundant as compared to the one of MAO-A isoform (Figures 5(a)–5(c)) in all groups. However, no difference in mRNA expression of both MAO isoforms could be detected among the groups (Figure 5(d)).

Despite the fact that MAO-A is considered the predominant isoform in cardiovascular system [17], relatively increased amounts of the MAO-B isoform have been reported in both rodent and human hearts (reviewed by Wang et al. [40]). In line with this observation, Kaludercic et al. were the first to demonstrate that an increased activity of MAO-B elicited mitochondrial dysfunction and structural/functional cardiac alterations in mice with experimentally induced heart failure [41]. Recently, we have also demonstrated the predominant expression of MAO-B in hearts and aortic rings harvested from diabetic animals [26].

### 3.6. MAO Protein Expression Was Comparable among the Groups

**Figure 6:**
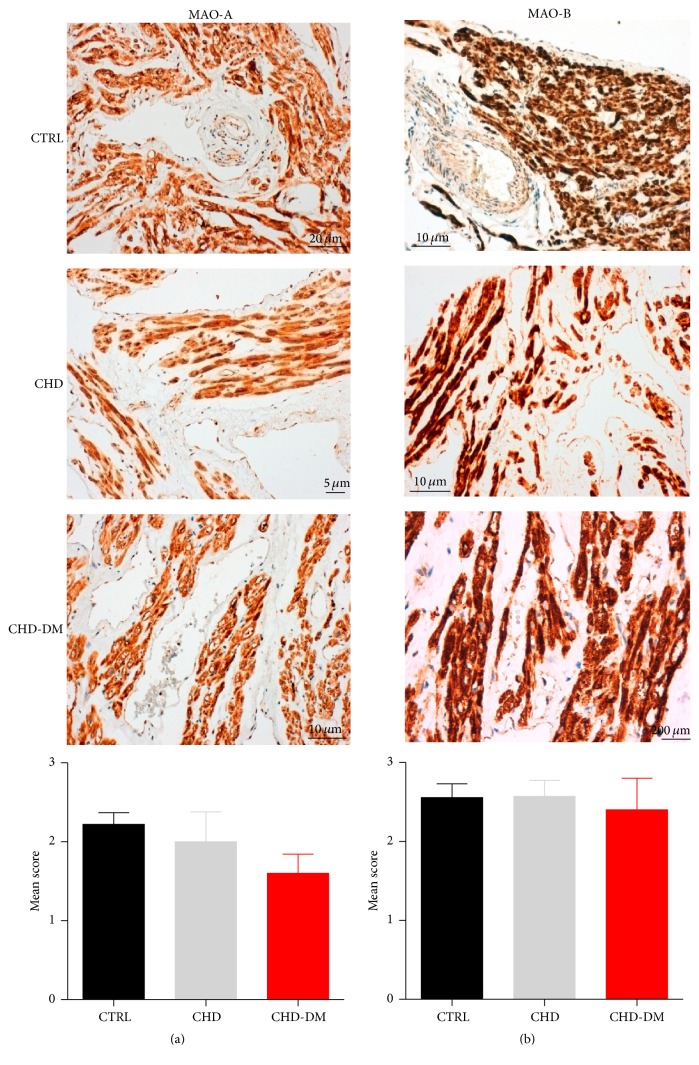
MAO protein expression in human atrial samples. The immunohistochemistry assay for MAO-A and MAO-B in atrial samples (a) and the corresponding results expressed as a mean score of intensity (b) (*n* = 10/group).

In order to assess whether mRNA induction also translates into changes in protein expression we further performed immunohistochemistry of the atrial samples. We observed the colocalization of MAO-A and MAO-B in the sarcoplasm of cardiomyocytes, fibroblasts, endothelial cells, and mesothelial cells, and the absence of the immunostaining in the collagen fibers of the connective tissue (Figure 6(a)). Both MAO isoforms were better expressed within the cardiac muscle cells, and again the MAO-B isoform was strongly expressed as compared to MAO-A in all three groups (Figure 6(a)). Thus, the previous results at gene level have been also confirmed at protein level (Figure 6(b)). Of note, we also found an increased expression of MAO-B protein in rodent hearts with streptozotocin-induced diabetes as compared to the nondiabetic animals [26].

The present study was purported to assess mitochondrial respiratory function and characterize the contribution of myocardial MAOs to the oxidative stress in diabetic coronary patients as compared to a group of nondiabetic coronary patients and of valvular patients with no documented CHD, respectively. Our findings confirm that diabetes elicits the impairment of mitochondrial respiratory function, regardless of the substrates used. A decreased oxidative phosphorylation and energy production in diabetic patients are suggestive for the hypothesis that mitochondrial dysfunction represents the underlying cause of diabetic cardiomyopathy (DCM) [42]. We showed here that both isoforms of MAO are expressed in atrial appendages harvested from patients with cardiovascular pathology regardless of the underlying disease, with the predominance of the MAO-B isoform. However, an important limitation of this study is that we did not investigate a possible link between mitochondrial dysfunction and MAOs activation. Indeed, Kaludercic et al. have recently demonstrated that MAO-B activation and inhibition of aldehyde dehydrogenase 2 activity contributed to altered mitochondrial bioenergetics [41].

We have also showed that* ex vivo* MAO-A and MAO-B inhibition significantly reduced ROS production, which strongly suggests a role for MAOs as contributors to oxidative stress in diseased atrial myocardium (namely, valvular diseases and coronary heart disease with/without diabetes). The subcellular targets of MAO-derived ROS have not been identified so far. This issue is particularly important, in light of the novel concepts of functional repair of oxidatively damaged proteins [43] and compartmentalization of oxidative stress [44].

Several other enzymatic systems, such as NADPH-oxidases (Nox) and xanthine oxidase, are recognized cellular sources of oxidative stress [45, 46]. In an early study we have shown that MAO inhibitors had no effect on xanthine oxidase-dependent H_2_O_2_ formation, and Nox activity in cells overexpressing Nox1, Nox2, and Nox4 was not affected by MAO inhibition [25]. Another relevant question is whether MAO inhibitors are not antioxidants* per se*. However, in the above-mentioned study, no significant antioxidant effect of MAO inhibitors was observed by two different H_2_O_2_ assays (Amplex red and FOX assay) [25].

We do acknowledge as further limitations of our study that (i) the antioxidant defence systems in the heart were not assessed (as possible reason for the lack of high oxidative stress in the setting of diabetes) and (ii) data from coronary patients were compared with those obtained from valvular patients (since a true “control group” is obviously difficult to recruit).

To date, there is only one study that identified MAO as an important determinant of redox balance in human atrial myocardium and associated MAO overexpression with an increased risk for postoperative atrial fibrillation [38]. However, experimental evidence available so far unequivocally demonstrated that monoamine oxidases contribute to mitochondrial and endothelial dysfunction, both widely investigated pathomechanisms and valuable therapeutic targets in cardiometabolic diseases. MAO inhibitors (MAOI) are currently used mainly in psychiatry and neurology. Given their already wide prescription for mood disorders, depression, and Parkinson's disease, the use of MAO inhibitors for cardiac disorders and diabetic cardiomyopathy should not present with major issues in terms of safety, at least at certain dosages. Nevertheless, translating basic science's predictions on the beneficial role of MAOI in the setting of heart failure and diabetes is likely to encounter resistance from medical community; the major concern is related to the risk for hypertensive crisis reported to occur with the first generation of irreversible MAOI when dietary tyramine is ingested. Of note, selegiline (the selective and irreversible MAO-B inhibitor) is also available as a transdermal patch; this type of administration clearly prevents the side-effects related to MAOI ingestion. However, whether this administration will achieve the circulating concentration relevant for the protective effects of the drugs in cardiovascular system remains to be investigated. Moreover, the novel selective and reversible MAOI (such as moclobemide) are reported to be both well-tolerated compounds and free from the above-mentioned interaction included in the elderly [47]. Of note, we have recently reported that moclobemide improved endothelial function impaired by* in vitro* incubation of isolated canine carotid arteries with angiotensin-II [48]. Whether this effect can be recapitulated after the* in vivo* administration of the drug remains to be demonstrated.

## 4. Conclusion

We report here the impairment of mitochondrial respiratory function in diabetic coronary patients regardless of the substrate used and the contribution of MAOs, in particular of the MAO-B isoform, to oxidative stress in human coronary hearts with and without DM. Further studies addressing the role of the novel selective and reversible MAO inhibitors in mitigating oxidative stress in cardiometabolic diseases are warranted.

## Figures and Tables

**Table 1:** Patients' demographic, clinical data, and preoperative medication.

Study groups	CTRL (*n* = 25)	CHD (*n* = 30)	CHD-DM (*n* = 20)
Demographics			
Age	63 ± 9.5	63 ± 8.5	61 ± 9
Sex, M/F (male/female)	15/10	26/4	16/4
Clinical characteristics			
BMI	26.7 ± 5.7	27 ± 4	30 ± 3.2^*∗*†^
Cholesterol (mg/dL)	184.8 ± 49.1	153.2 ± 33^*∗*^	148 ± 23^*∗*^
FPG	103.3 ± 20.2	105 ± 16	192 ± 64^*∗*†^
LVEF	55.7 ± 7.8	48.5 ± 8.4^*∗*^	48 ± 7.7^*∗*^
HT	15 (60)	30 (100)^*∗*^	20 (100)^*∗*^
AF	3 (12)	2 (6.67)	0
Preoperative medication			
Aspirin	5 (20)	30 (100)	20 (100)
*β*-blockers	20 (80)	30 (100)	20 (100)
Anticoagulants	25 (100)	30 (100)	20 (100)
Statins	18 (72)	30 (100)	20 (100)
Nitrates	8 (32)	21 (70)	15 (75)
Calcium channel blockers	5 (20)	8 (26.67)	7 (35)
ACE inhibitors	0	30 (100)	20 (100)
Diuretics	25 (100)	30 (100)	20 (100)
Insulin	0	0	5 (25)
Oral antidiabetics	0	0	20 (75)
Antibiotherapy	25 (100)	30 (100)	20 (100)

Data are means ± SEM. In parentheses the percentages of the corresponding variable are mentioned. ^*∗*^ *p* < 0.05 versus CTRL; ^†^ *p* < 0.05: CHD versus CHD-DM. BMI: body mass index, FPG: fasting plasma glucose, LVEF: left ventricular ejection fraction, HT: hypertension, AF: atrial fibrillation, and ACEI: angiotensin-converting-enzyme inhibitors.
